# DLL3: an emerging target in small cell lung cancer

**DOI:** 10.1186/s13045-019-0745-2

**Published:** 2019-06-18

**Authors:** Dwight H. Owen, Michael J. Giffin, Julie M. Bailis, Marie-Anne Damiette Smit, David P. Carbone, Kai He

**Affiliations:** 1 Division of Medical Oncology, Department of Internal Medicine, The Ohio State University Comprehensive Cancer Center, Columbus, OH USA; 2 Oncology Research, Amgen Inc., Thousand Oaks, CA USA; 3 Oncology Research, Amgen Inc., South San Francisco, CA USA; 4 Translational Medicine, Amgen Inc., Thousand Oaks, CA USA

**Keywords:** Antibody-drug conjugate (ADC), Bispecific T cell engager (BiTE®) antibody construct, Chimeric antigen receptor (CAR) T cell therapy, Delta-like ligand 3 (DLL3), Immuno-oncology therapy, Neuroendocrine, Small cell lung cancer (SCLC), Targeted therapy

## Abstract

Small cell lung cancer (SCLC) accounts for approximately 15% of all lung cancers. Despite high rates of response to first-line chemotherapy and radiotherapy, patients with extensive-stage disease eventually relapse, and very few patients survive more than 5 years from diagnosis. Treatment options for recurrent or refractory disease are limited, and the treatments that do exist are associated with significant treatment-related toxicities. Delta-like ligand 3 (DLL3) is an inhibitory Notch ligand that is highly expressed in SCLC and other neuroendocrine tumors but minimally expressed in normal tissues. It is therefore being explored as a potential therapeutic target in SCLC. Here, we review the preclinical and clinical evidence for targeting DLL3 in SCLC and discuss several DLL3-specific therapies being developed for the treatment of SCLC: the antibody-drug conjugate rovalpituzumab tesirine, the bispecific T cell engager immuno-oncology therapy AMG 757, and the chimeric antigen receptor T cell therapy AMG 119.

## Background

Lung cancer is the most common cause of cancer death, and small cell lung cancer (SCLC) represents approximately 15% of all cases [1]. Despite remarkable progress in the treatment of non-small cell lung cancer in the last decade [2], patients with SCLC continue to have a poor prognosis and limited treatment options [3]. Recently, the addition of the anti-PD-L1 antibody atezolizumab (TECENTRIQ®) to carboplatin and etoposide chemotherapy demonstrated an improvement in overall survival (OS) in the first-line setting (median OS of 12.3 months versus 10.3 months for chemotherapy alone [95% confidence interval, 0.54–0.91; *P* = 0.007]), leading to the approval of this regimen by the United States Food and Drug Administration (FDA) for first-line treatment of extensive-stage SCLC [4, 5]. Although the approval of atezolizumab for first-line treatment marks an important step forward in the treatment of SCLC, the limited 2-month benefit highlights the need for development of additional therapies.

Treatment of SCLC beyond the first line is still associated with low response rates despite decades of clinical trials [6–9]. The anti-PD-1 antibody nivolumab was recently granted accelerated approval by the FDA for the treatment of patients with SCLC with progression after at least two lines of chemotherapy, including one that contains platinum [10]. This approval was based on the results of the CheckMate-032 study (NCT01928394), an open-label study of nivolumab or nivolumab plus ipilimumab in SCLC [11]. However, a recent report indicates that nivolumab failed to improve OS compared to topotecan or amrubicin in a second-line SCLC patient population (CheckMate-331, NCT02481830) [12]. A randomized, multicenter, double-blind, phase 3 study of nivolumab, nivolumab in combination with ipilimumab, or placebo as maintenance therapy in patients with extensive-stage disease SCLC after completion of platinum-based first-line chemotherapy (CheckMate-451, NCT02538666) also failed to meet its primary endpoint of OS [13]. National Comprehensive Cancer Network (NCCN) Guidelines® recommend treatment with the original platinum-based doublet for recurrent disease that occurs greater than 6 months from treatment, while enrollment into a clinical trial is preferred for patients with recurrence less than 6 months from initial treatment given the limited clinical benefit observed with topotecan as well as the other agents studied in this setting [7, 14–16]. Therefore, identifying new treatment pathways for patients, especially in the second-line setting and beyond, is an area of dire clinical need.

The Notch pathway is a highly conserved cell-cell signaling pathway involved in a variety of development processes, including the development of pulmonary neuroendocrine cells [17, 18]. Delta-like ligand 3 (DLL3) is an inhibitory Notch pathway ligand that is highly upregulated and aberrantly expressed on the cell surface in SCLC and other high-grade neuroendocrine tumors [19, 20]. Notch signaling is downregulated during neuroendocrine tumor growth and is inhibited by DLL3 expression [20–22]. DLL3 expression is regulated by achaete-scute homolog 1 (ASCL1), a transcription factor that is required for proper development of pulmonary neuroendocrine cells and is an oncogenic driver in SCLC [23, 24]. In preclinical models, DLL3 expression promotes SCLC migration and invasion through a mechanism that involves control of the epithelial-mesenchymal transition protein Snail [25].

DLL3 is specifically expressed on the surface of SCLC tumor cells. DLL3 surface expression correlated with time to tumor progression in 10 SCLC and 1 large cell neuroendocrine carcinoma (LCNEC) patient-derived xenograft models [20]. In a study of 63 patients with SCLC, 52 (83%) patient tumor samples were found to be positive for DLL3 expression by immunohistochemistry (IHC), and 20 (32%) showed high expression of DLL3 (positive in at least 50% of cancer cells) [26]. Overall, greater than 80% of SCLC tumors express DLL3 mRNA and protein, and cytoplasmic and membranous staining of DLL3 was observed by IHC with a high level of homogeneity across neoplastic cells. In contrast, only a few normal cell types expressed DLL3 (e.g., neurons, pancreatic islet cells, and pituitary cells), and expression of DLL3 was exclusively cytoplasmic [27–29]. Recent studies have reported that DLL3 is also expressed in other tumor types of neuroendocrine origin, including melanoma, glioblastoma multiforme, small cell bladder cancer, metastatic castration-resistant prostate cancer, and neuroendocrine lung tumors [30–34].

The DLL3 expression profile—high, homogeneous cell surface expression in tumors, versus low, cytoplasmic expression in a subset of normal tissues—has enabled the development of therapeutics that use DLL3 to specifically target SCLC cells [20, 35]. These DLL3-specific agents are now being evaluated in several ongoing clinical studies in SCLC and other neuroendocrine tumors. In this review, we focus on the preclinical and clinical data supporting the development of novel therapies that target DLL3 in SCLC: the antibody-drug conjugate (ADC) rovalpituzumab tesirine, the bispecific T cell engager (BiTE®) immuno-oncology therapy AMG 757, and the chimeric antigen receptor (CAR) T cell therapy AMG 119 (Table 1, Table 2).

**Table 1:** Select ongoing clinical trials of DLL3-targeted agents in SCLC

Treatment	Setting	Primary outcome	Phase	*N*	ID
Rovalpituzumab tesirine, cisplatin, etoposide	First line	Safety and PFS	1	28	NCT02819999
Rovalpituzumab tesirine, nivolumab, ipilimumab	Second line	Safety	1/2	42	NCT03026166
Rovalpituzumab tesirine versus topotecan	Second line	OS	3	444	NCT03061812
Rovalpituzumab tesirine, dexamethasone	Maintenance	PFS, OS	3	740	NCT03033511
AMG 757	Second line	Safety, tolerability, and PK	1	92	NCT03319940
AMG 119	Second line	Safety and tolerability	1	41	NCT03392064

*MTD* maximum tolerated dose, *OS* overall survival, *PFS* progression-free survival, *PK* pharmacokinetics, *RP2D* recommended phase 2 dose, *SCLC* small cell lung cancer. Detailed information relevant to these clinical trials can be found at http://www.clinicaltrials.gov

**Table 2:** Clinical trials of DLL3-targeted therapies in SCLC that have been completed

Treatment	Setting	Primary objective	Phase	*N*	ID	Results	Ref.
Rovalpituzumab tesirine	SCLC, LCNEC	Safety	1/2	82 (74 SCLC)	NCT01901653	ORR, 18% (11/60)	[36]
Rovalpituzumab tesirine	SCLC, 3rd line, and beyond	ORR, OS	2	339	NCT02674568	ORR, 12.4% (9.1, 16.4); median OS, 5.6 months (4.9, 6.1)	[37]

*LCNEC* large cell neuroendocrine cancer, *ORR* overall response rate, *OS* overall survival, *SCLC* small cell lung cancer

## A DLL3-targeted ADC in SCLC

### Preclinical studies

**Fig. 1:**
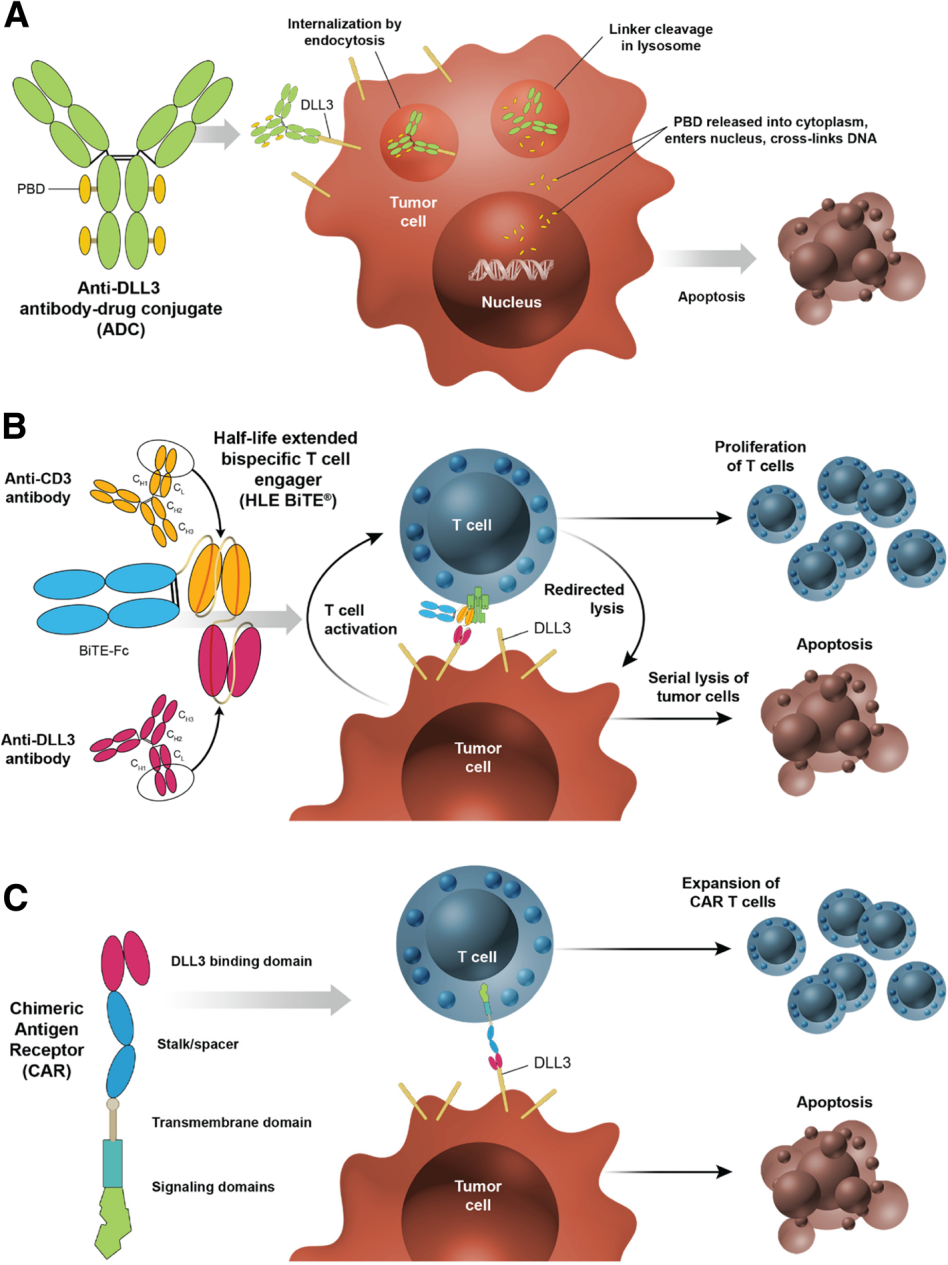
DLL3-targeted investigational products utilize distinct mechanisms of action. **a** Rovalpituzumab tesirine is a DLL3-targeted antibody-drug conjugate (ADC) that consists of a humanized DLL3-specific IgG1 monoclonal antibody, a pyrrolobenzodiazepine (PDB) dimer toxin, and a protease-cleavable linker that covalently links the antibody to the toxin. Internalization of the ADC to lysosomes leads to the cleavage of the linker, release of the toxin, and apoptosis. **b** AMG 757 is a half-life extended bispecific T cell engager (HLE BiTE®) antibody construct that consists of a single-chain (sc) Fv domain that binds DLL3, an scFv domain that binds CD3ε (an invariable part of the T cell receptor complex), and a fragment crystallizable (Fc) region. AMG 757 is designed to transiently connect DLL3-positive cells to CD3-positive T cells and induce serial lysis of tumor cells and concomitant proliferation of T cells. **c** AMG 119 is an adoptive cellular therapy that consists of a patient’s own T cells that have been genetically modified ex vivo to express a chimeric antigen receptor (CAR) that targets DLL3 and redirects cytotoxic T cells to DLL3-positive cells. AMG 119 is designed to expand and persist in vivo and induce apoptosis of tumor cells

ADCs use an antibody against a tumor cell surface antigen to deliver chemotherapy to tumor cells and enable internalization of the compound to promote tumor cell killing (Fig. 1). A novel DLL3-targeted ADC, SC16DL6.5, demonstrated durable responses in SCLC and LCNEC patient-derived xenograft (PDX) preclinical models [20]. This DLL3-ADC, now known as rovalpituzumab tesirine, is composed of a humanized DLL3-specific IgG1 monoclonal antibody, the DNA cross-linking agent pyrrolobenzodiazepine (PDB), and a protease-cleavable linker [20]. In immunofluorescence colocalization studies, rovalpituzumab tesirine was internalized to late endosomes by DLL3-expressing cells. In PDX studies, mice treated with rovalpituzumab tesirine had rapid and prolonged responses compared to mice treated with the standard-of-care chemotherapy cisplatin and etoposide [20]. Even PDX tumors that were resistant to chemotherapy or recurred following cisplatin and etoposide showed responses to rovalpituzumab tesirine, including complete responses [20].

### Clinical experience

Based on the preclinical data detailed above, rovalpituzumab tesirine was evaluated in a phase 1 study [36]. NCT01901653 was a first-in-human, open-label, phase 1 study of rovalpituzumab tesirine in patients with recurrent or progressive SCLC or LCNEC to determine safety, tolerability, and maximum tolerated dose (MTD). The study included 82 patients at 10 cancer centers within the USA, of whom 74 had SCLC and 8 had LCNEC. The median age of patients was 61 years, 42 (57%) were male, 35 (47%) had received two prior lines of therapy, and 21 (28%) had a history of central nervous system (CNS) metastases. The study enrolled patients to dose-escalation or dose-expansion cohorts at doses between 0.05 and 0.8 mg/kg, on either a once-every-3-weeks (Q3W) or once-every-6-weeks (Q6W) schedule. Intrapatient dose escalation was not allowed. Endpoint analyses were restricted to only the 74 patients with SCLC. The MTD was determined to be 0.4 mg/kg Q3W. Dose-limiting toxicities observed included grade 4 thrombocytopenia and grade 4 liver function test changes, which were reversible. Unfortunately, dosing patients at the MTD was found to have the unacceptable delayed toxicity of serosal effusion (including pleural and pericardial effusion). Pharmacokinetic studies revealed that the half-life of rovalpituzumab tesirine was 10–14 days, so additional dosing schedules were explored. Ultimately, the dose regimens of 0.2 mg/kg Q3W or 0.3 mg/kg Q6W were explored in expansion cohorts, with a maximum capped total dose of 0.6 mg/kg (i.e., 3 doses of 0.2 mg/kg Q3W or 2 doses of 0.3 mg/kg Q6W). The dosing regimen of 0.3 mg/kg Q6W was recommended for evaluation in further clinical trials [36].

From the phase 1 study, a unique pattern of toxicities emerged. For the 74 patients with SCLC, treatment-related adverse events of any grade occurred in 65 (88%) patients, with toxicities of grade 3 or higher occurring in 28 (38%) patients. The most frequent adverse events of grade 3 or higher were thrombocytopenia (8 patients, 11%), pleural effusion (6 patients, 8%), and elevated lipase (5 patients, 7%). Grade 3 or higher serosal effusions occurred in 8 (11%) patients and included pleural and pericardial effusions as well as capillary leak syndrome. Various grade 3 or higher skin reactions, ranging from maculopapular rash to erythema multiforme and palmar-plantar erythrodysesthesia, occurred in 6 (8%) patients. Pleural effusion of any grade occurred in 23 (31%) patients, and pericardial effusion of any grade occurred in 9 (12%) patients. In the entire cohort of 82 patients, treatment with rovalpituzumab tesirine was withdrawn due to adverse events in 18 (22%) patients. While the source of these toxic effects is not clear, they may be related to the PDB dimer portion of the rovalpituzumab tesirine ADC [36]. Two treatment-related deaths occurred during the study: one due to endobronchial tumor hemorrhage associated with tumor progression and marked thrombocytopenia after 10 days on study, and another due to acute renal injury after 128 days on study, which was thought to be related to nephrotoxic agents used to manage drug-related peripheral edema.

Among the 74 patients with SCLC who received any dose of rovalpituzumab tesirine, 65 were assessable for activity. Of these patients, 11 (17%) achieved a confirmed objective response (complete response or partial response) and 35 (54%) had stable disease. Of the 60 patients who received active doses (0.2 mg/kg or 0.4 mg/kg Q3W or 0.3 mg/kg or 0.4 mg/kg Q6W), 11 (18%) had a confirmed objective response and 30 (50%) had stable disease. In an exploratory analysis of 39 patients who provided tumor samples for analysis of DLL3 expression, 29 assessable patients had DLL3-high tumors (defined as expression in 50% or more tumor cells by IHC), and 10 (35%) of these patients had a confirmed objective response. None of the 10 patients with DLL3-low tumors (defined as expression in less than 50% tumor cells by IHC) had a response to treatment. For the 68 patients treated with active doses of rovalpituzumab tesirine, median OS was 4.6 months (95% CI, 3.9–7.1).

TRINITY (NCT02674568) was an open-label, single-arm, phase 2 study of rovalpituzumab tesirine in patients with DLL3-expressing SCLC (defined as expression in 1% or more tumor cells by IHC) in the second-line setting or beyond. Data from this study was presented at the 2018 American Society of Clinical Oncology (ASCO) Annual Meeting [37]. The study included 339 patients treated with rovalpituzumab tesirine at a dosing schedule of 0.3 mg/kg Q6W for two doses, with retreatment permitted upon progression. With a median follow-up of 19.1 weeks (range 0.6–90.6 weeks) at the time of presentation, the overall response rate was 18.0% (95% CI, 14.1–22.5) for all patients per investigator assessment and 12.4% (95% CI, 9.1–16.4) by independent review. The median OS was 5.6 months (95% CI, 4.9–6.1), and 66% of patients completed the planned two doses of rovalpituzumab tesirine. Treatment of patients with DLL3-high tumors (defined in this case as expression in more than 75% of tumor cells by IHC) did not result in significantly different OS or response rates compared to all dosed patients. Toxicities in the phase 2 study were consistent with the findings of the phase 1 study. Grade 3/4 treatment-related adverse events included thrombocytopenia in 37 (11%) patients, photosensitivity reaction in 23 (7%) patients, and pleural effusion in 14 (4%) patients. Overall, drug-related adverse events of any grade occurred in 308 (91%) patients, with 134 (40%) patients having grade 3 or higher toxicities. Ten (3%) patients had fatal drug-related adverse events, including generalized edema (*n* = 2), pneumonitis (*n* = 2), ascites (*n* = 1), drug-induced liver injury (*n* = 1), pleural effusion (*n* = 1), pneumothorax (*n* = 1), respiratory failure (*n* = 1), and sepsis (*n* = 1).

Unfortunately, the phase 3 trial comparing rovalpituzumab tesirine to topotecan as second-line therapy for SCLC (TAHOE, NCT03061812) was recently halted after the Independent Data Monitoring Committee recommended stopping enrollment due to shorter OS in the rovalpituzumab tesirine arm compared with the topotecan arm [38]. The adverse findings with rovalpituzumab tesirine may be related to the DNA cross-linking agent PBD, which is the cytotoxic payload of the ADC [36]. While the rovalpituzumab tesirine molecule induced potent antitumor activity in vitro and in mouse models, it may be necessary to modify the molecule, e.g., through addition of an alternative cytotoxic payload or use of alternate linker chemistry, to achieve acceptable tolerability. Alternatively, exploration of a different dose and schedule of rovalpituzumab tesirine in other disease settings may identify opportunities for clinical development. Consistent with this hypothesis, a study of rovalpituzumab tesirine as maintenance therapy following first-line platinum-based chemotherapy (MERU, NCT03033511) continues to enroll. Despite the cessation of the TAHOE trial, DLL3 remains a high-value target in SCLC due to its high, homogeneous expression on the surface of tumor cells and its low, relatively restricted, cytoplasmic expression in normal cells.

## DLL3-targeted BiTE® molecules and CAR T cells in SCLC

### Preclinical studies

Immunotherapies based on redirected T cell cytotoxicity, including BiTE® molecules and CAR T cells, provide a novel approach that utilizes cells of the immune system to target DLL3-expressing tumor cells (Fig. 1). Based on preclinical data, both BiTE® molecules and CAR T cells have the potential for direct cell killing of DLL3-positive SCLC tumor cells, even at low levels of DLL3 cell surface expression (< 1000 receptors per cell) [39]. The improvement in OS with the addition of atezolizumab to chemotherapy in the first-line setting confirms the utility of immunotherapy in this patient population. In addition, the tumor responses seen after treatment with rovalpituzumab tesirine validate DLL3 as a target. BiTE® molecules and CAR T cells would not be expected to show the same toxicity profile as rovalpituzumab tesirine because they do not contain a cytotoxic payload.

Bispecific binding of the BiTE® molecule to a tumor-associated antigen on target cells and CD3 on endogenous T cells leads to formation of a cytolytic synapse and results in antigen-dependent target cell lysis, T cell activation, and cytokine production [40]. BiTE® molecule activity does not require a specific T cell receptor or peptide-MHC complex and may have the potential to overcome the immunosuppressive environment of tumors [40]. Clinical validation of the BiTE® immuno-oncology platform was achieved with the anti-CD19 x CD3 BiTE® molecule blinatumomab (Blincyto®), which received accelerated approval from the FDA in December 2014 for the treatment of B cell precursor acute lymphoblastic leukemia (B-ALL) [41, 42].

BiTE® molecules that target DLL3 have been generated and characterized in vitro and in vivo. AMG 757 is an anti-DLL3 x CD3 BiTE® antibody construct that is fused to an Fc domain to allow an extended pharmacokinetic half-life. In T cell-dependent cytotoxicity assays performed with SCLC cell lines in vitro, low picomolar concentrations of AMG 757 were able to redirect T cells to kill DLL3-positive cancer cells. AMG 757 potency was maintained in these assays even against cell lines that express low levels of cell surface DLL3 protein (< 1000 molecules per cell). AMG 757 had no effect on cells that did not express cell surface DLL3. Consistent with the BiTE® mechanism of action, AMG 757 induces T cell activation and cytokine production when T cells are incubated in vitro with DLL3-positive SCLC cells [35, 39, 43].

In a disseminating orthotopic model of SCLC, tumor regression was observed in vivo with low milligram-per-kilogram weekly doses of AMG 757. In this model, SHP-77 cells were injected by intravenous (IV) injection into immunocompromised NOD *scid* gamma (NSG™) mice. The cells then migrated to the lung and formed tumors. Mice bearing an established tumor in the lung were administered a single dose of human T cells and a once weekly intraperitoneal dose of AMG 757. This model recapitulated the biologic compartment for primary SCLC tumors by requiring that both T cells and BiTE® molecules traffic to the lung tumor to achieve efficacy [35, 39].

The pharmacokinetic properties of AMG 757 were evaluated in non-human primates. The half-life of AMG 757 in this nonclinical model was greater than 200 h, and pharmacokinetic modeling projected that AMG 757 may be dosed once weekly or less frequently in humans. In nonclinical toxicology studies, AMG 757 was well tolerated up to doses of 4.5 mg/kg, consistent with the limited expression of DLL3 in normal tissues [35, 39].

An alternative strategy to harness a patient’s T cells for cancer therapy is the use of CAR T cells. These are T cells that are taken from a patient and genetically modified to express a receptor for a tumor antigen. Following modification, they are re-administered to the patient for cancer therapy. CAR T cells then target tumor cells that express the antigen of interest and undergo activation and expansion that enables tumor cell killing. Clinical validation of the CAR T platform was achieved with two CAR T products that are engineered to express receptors for CD19. Both have been approved by the FDA. Tisagenlecleucel (Kymriah®) is approved for B-ALL and large B cell lymphoma [44–47], and axicabtagene ciloleucel (Yescarta®) is approved for large B cell lymphoma [48–50].

AMG 119 is an adoptive cellular therapy that consists of autologous T cells that are genetically modified ex vivo to express a transmembrane chimeric antigen receptor that targets DLL3. In contrast to the AMG 757 BiTE® molecule, the AMG 119 CAR T cells have the potential to achieve prolonged antitumor activity with a single administration. AMG 119 shows potent eradication of DLL3-positive cells in vitro, with robust ablation of target cells at all levels of DLL3 expression tested, including expression of < 1000 DLL3 molecules per cell as measured by flow cytometry [35, 39, 43]. This DLL3-dependent cytotoxic activity is accompanied by production of pro-inflammatory cytokines, consistent with the mechanism of T cell-mediated cytotoxicity and antigen-dependent T cell activation. Furthermore, co-culture of AMG 119 with DLL3-positive target cells results in the proliferation of the engineered T cells, suggesting that the antigen-dependent signaling pathways remain intact and functional. In vivo, a single administration of AMG 119 reduced mean tumor volume in a SHP-77 xenograft model [35, 39, 43]. These preclinical data suggest that AMG 119 may have high potency and specificity for DLL3-positive SCLC tumor cells.

### Clinical experience

Both AMG 757 and AMG 119 are currently being investigated in first-in-human studies. NCT03319940 is an open-label, phase 1 study evaluating the safety, tolerability, and pharmacokinetics of AMG 757 administered as an IV infusion once every 2 weeks [51]. The study will initially enroll adult patients with relapsed/refractory SCLC who have progressed or recurred following platinum-based chemotherapy. Additional inclusion criteria include Eastern Cooperative Oncology Group (ECOG) performance status 0–2, minimum life expectancy of 12 weeks, at least 2 measurable lesions per modified response evaluation criteria in solid tumors (RECIST) 1.1 criteria, no untreated or symptomatic brain metastases, and adequate organ function. The study will later enroll patients with extensive disease SCLC with ongoing clinical benefit following no more than 6 cycles of first-line platinum-based chemotherapy.

NCT03392064 is an open-label, phase 1 study evaluating the safety, tolerability, and efficacy of AMG 119 in adult patients with SCLC whose disease has progressed or recurred after at least one platinum-based regimen. Key inclusion criteria include ECOG performance status 0–1, at least two measurable lesions per modified RECIST 1.1 criteria, no untreated or symptomatic brain metastases, and adequate organ function. AMG 119 will be administered as a one-time IV infusion.

## Conclusion

SCLC is a devastating disease with a poor prognosis. Few therapeutic advances have been made over the last several decades, but recently, first-line treatment with atezolizumab in addition to chemotherapy demonstrated an improvement in OS [4, 5]. While addition of an anti-PD-L1 antibody to first-line treatment may benefit many patients, it may limit the development and use of anti-PD-1(L1) agents in second or later lines. The approval of atezolizumab and subsequent changes to the standard of care may also result in challenges to the conduct and interpretation of ongoing clinical trials in the first-line and the maintenance settings. Treatment options for patients in the second line and beyond remain limited, highlighting the need for development of additional therapies.

Rovalpituzumab tesirine, a DLL3-targeted ADC, has shown early signs of efficacy, even in patients in the third- and fourth-line settings. However, the unique toxicity profile of rovalpituzumab tesirine, which appears to be related to the DNA cross-linking agent PBD [36], might limit its clinical utility. DLL3 nonetheless remains a promising target. DLL3 is highly expressed in SCLC and other neuroendocrine tumors, and it has low to no expression in most normal tissues. Targeting DLL3 through T cell-redirecting therapies may be an alternative way of treating DLL3-positive tumors. Ongoing studies with rovalpituzumab tesirine and with immuno-oncology therapies such as AMG 757 and AMG 119 are expected to provide us with a better understanding of the potential of this novel target and perhaps finally provide patients with more effective treatment options for this very aggressive disease.

## Data Availability

The material supporting the conclusion of this review has been included within the article.

## Abbreviations

ADC: Antibody-drug conjugate
BiTE®: Bispecific T cell engager
CAR T: Chimeric antigen receptor T cells
DLL3: Delta-like ligand 3
ECOG: Eastern Cooperative Oncology Group
IHC: Immunohistochemistry
IV: Intravenous
LCNEC: Large cell neuroendocrine carcinoma
ORR: Overall response rate
OS: Overall survival
PFS: Progression-free survival
RECIST: Response evaluation criteria in solid tumors
SCLC: Small cell lung cancer
